# Effects of School Closures, 2008 Winter Influenza Season, Hong Kong

**DOI:** 10.3201/eid1410.080646

**Published:** 2008-10

**Authors:** Benjamin J. Cowling, Eric H.Y. Lau, Conrad L.H. Lam, Calvin K.Y. Cheng, Jana Kovar, Kwok Hung Chan, J.S. Malik Peiris, Gabriel M. Leung

**Affiliations:** University of Hong Kong, Hong Kong Special Administrative Region, People’s Republic of China (B.J. Cowling, E.H.Y. Lau, C.L.H. Lam, C.K.Y. Cheng, K.H. Chan, J.S.M. Peiris, G.M. Leung); University College London, London, UK (J. Kovar)

**Keywords:** human influenza, sentinel surveillance, public health, school closures, social distancing, primary school, kindergarten, dispatch

## Abstract

In winter 2008, kindergartens and primary schools in Hong Kong were closed for 2 weeks after media coverage indicated that 3 children had died, apparently from influenza. We examined prospective influenza surveillance data before, during, and after the closure. We did not find a substantial effect on community transmission.

Hong Kong, Special Administrative Region, People’s Republic of China, is a subtropical city in Southeast Asia at the epicenter of global influenza activity (*1*). Epidemiologically, influenza usually displays biannual seasonality, consisting of a winter peak typically between January and March and a summer peak in June and July, often with an elevated plateau in April and May between the winter and summer peaks (*2*–*4*).

On the evening of March 12, 2008, after 3 children had died, apparently from influenza, the government of Hong Kong announced that all primary schools, special schools, kindergartens, and day nurseries would close the following day for a total of 2 weeks, 1 week earlier than the scheduled start of the annual week-long Easter break (*5*,*6*).

## The Study

We reviewed prospective surveillance data on influenza and influenza-like illness activity during the 2008 winter influenza season. We then considered the effects of the school closures on community transmission.

As elsewhere in the Northern Hemisphere, the 2007–08 strains of influenza virus circulating in Hong Kong were closely related to A/H1N1/Brisbane/59/2007, A/H1N1/Soloman Islands/3/2006-like, A/H3N2/Brisbane/10/2007, B/Yamagata/16/88-like, and B/Malaysia/2506/2004-like. These strains were not well matched to the trivalent inactivated vaccine specified for the season.

Surveillance data from different settings before, during, and after the period of school closures are shown in the Figure. Laboratory isolation of influenza viruses in children (panel A) and adults (panel B) show that the influenza season began in January, rose to a peak in late February, and was already waning by the time the decision was made to close schools, as temperatures and relative humidity were increasing (*8*). Influenza circulation has remained at a low baseline level since schools reopened in early April. Absenteeism rates in sentinel childcare centers and primary schools gradually rose to maximums of 7.9% and 3.5%, respectively, before the school closures and returned to low levels after the closures (data not shown). Similarly, influenza consultation rates at public and private outpatient clinics (panel C) peaked before the closures and generally reflected the reference laboratory data, except for a dip during Chinese New Year, when many sentinel practices were closed.

When compared with the influenza seasons of the preceding 9 years, the 2008 winter influenza season was moderately severe in terms of outpatient consultations (Appendix Figure). Influenza hospital admission rates in children <4 years reached peak levels of 30/100,000 population in 2006 and 41/100,000 in 2007, both mild seasons. These rates were similar to the peak level of 39/100,000 in 2008 (Figure, panel D) (*6*). The elderly appeared to have been less affected, with no clear rate increases noted by febrile sentinel surveillance in elderly care homes and generally low influenza-related admission rates in this age group (data not shown).

Panel E of the Figure shows daily estimates of the effective reproductive number, or *R_t_*, based on a simple method (*9*) that we applied to daily interpolations of the laboratory and outpatient sentinel data. We used a Weibull model for the serial interval with mean of 3.6 days and standard deviation of 1.6 days, based on data from a recent community study (*10*). The effective reproductive number on day *t* can be interpreted as the average number of new persons infected by an infector who had symptom onset on day *t.* Therefore, a reproductive number >1 implies that an epidemic will grow in the short term, whereas a number <1 implies that an epidemic will die out. These trends, in particular the lack of any apparent negative inflection point during the entire 2-week period of school closure, suggest that the effect of the intervention was not substantial. Trends in estimated *R_t_* were similar if serial intervals of mean 2.5 or 2.0 days were assumed.

## Conclusions

Although we can only speculate, given the limitations of an uncontrolled natural experiment on the population level, routine surveillance data did not detect a large effect from the school closures. In particular, we noted a decline in laboratory isolations of influenza viruses that preceded the intervention and the lack of association between school closures and *R_t_*. In fact, sentinel data may not accurately represent the incidence of influenza in the underlying population because, for example, other cocirculating upper respiratory viruses contribute to overall influenza-like illness consultation rates. Laboratory data, however, should be less affected, and extra testing in response to the heightened awareness of influenza activity might have artifactually lowered the positivity rate. The epidemic curves generated from the surveillance data showed a decline in cases that may have naturally concluded without any intervention. We note the difficulty of making inferences directly from changes in epidemic curves because changes in the epidemic curve may lag behind changes in the underlying transmission dynamics by at least 1 serial interval, as has previously been shown for severe acute respiratory syndrome (*9*,*11*). Although the estimates of *R_t_* (panel E) are crude, the estimated values of 1.2–1.5 during the rising phase of the 2008 winter epidemic in Hong Kong are slightly lower than previous estimates for interpandemic influenza (*12*,*13*), perhaps because of the low time-dependent resolution of the weekly aggregation of surveillance data.

We emphasize that our results must be interpreted with caution; in particular, influenza might have continued to circulate for a longer period had the school closures not been implemented. Furthermore, notwithstanding our tentative null findings, some previous reports have demonstrated that school closures may be effective at mitigating influenza seasons. For example, a study showed significant reduction in respiratory infections during school closures in Israel (*14*), and a recent model estimated that school holidays prevent 16%–18% of seasonal influenza cases in France (*12*).

We acknowledge that our assessment has the benefit of hindsight, whereas at the time the decision was made to close schools it might well have been unclear from surveillance data that the influenza season was only moderate and might have already been in natural decline. Although daily hospital admissions data were available in real time from a new integrated computer system and therefore did show the decline, this system only reflected serious illness. However, outpatient sentinel data, which are more indicative of overall influenza activity in the general community, were available with an ≈7-day lag; reports of laboratory reference data lagged even further. If public health decisions are to be made on the basis of prospective surveillance, these systems must be improved to reflect real-time or near real-time reporting and analysis. One possibility in Hong Kong would be to use the wealth of data from rapid influenza tests in hospitals, now that >1,000 rapid tests are conducted every month on most newly admitted patients with pneumonia or respiratory symptoms. Furthermore, although most local surveillance data are aggregated (Figure), the spread of influenza likely varies according to population subgroup. For example, influenza infections in children cause considerable illness and death, and it is often hypothesized that children are affected generally earlier in epidemics because of the higher transmission rates (*15*). Therefore, justification is strong for local authorities to begin collecting and reporting timely age-specific community surveillance in sentinel and laboratory networks.

**Figure:**
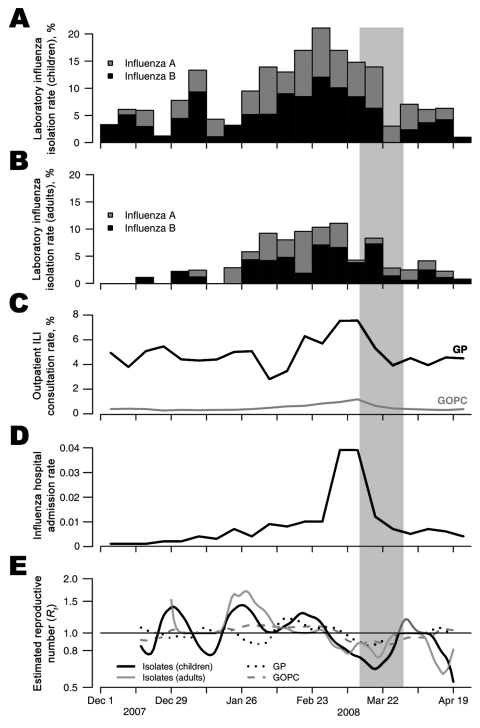
Influenza surveillance data from December 1, 2007, through April 26, 2008, including the 2-week school closure period (gray vertical bar): A) Proportion of influenza A and B isolations (by date of collection) among all children’s specimens that were submitted to the World Health Organization (WHO) reference laboratory at Queen Mary Hospital (most specimens are referred from local hospitals). B) Proportion of influenza A and B isolations (by date of collection) among all adult patients’ specimens that were submitted to the WHO reference laboratory at Queen Mary Hospital. C) Weekly influenza-like illness (ILI) (defined as fever plus cough or sore throat) consultation rates in sentinel networks of outpatient clinics in the private (GP) and public (GOPC) sectors. D) Weekly rates of public hospital admissions in young children (<4 years) with a principal diagnosis of influenza (International Classification of Diseases, 9th revision, code 487), where the denominator is the general population of the same age. E) Daily estimates of the effective reproductive number based on the laboratory and sentinel outpatient data. Source for panels B–D (*7*).

## Supplementary Material

Appendix Figure Weekly influenza-like illness (ILI) consultation rates in sentinel networks of outpatient clinics in the private (GP) and public (GOPC) sectors between December 1988 and March 2008 (lines) and monthly proportions of influenza A and B isolations among all specimens submitted to the Public Health Laboratory of the Department of Health of the Hong Kong Special Administrative Region, People's Republic of China, government (bars); the vertical superimposed bars indicate school holidays at Christmas, Chinese New Year, and Easter. Source: (*7*).
