# Supplementary figures and images for: Effects of School Closures, 2008 Winter Influenza Season, Hong Kong
Source: Emerg Infect Dis. 2008 Oct;14(10):1660–2. doi: 10.3201/eid1410.080646 (PMC2609897; doi:10.3201/eid1410.080646)

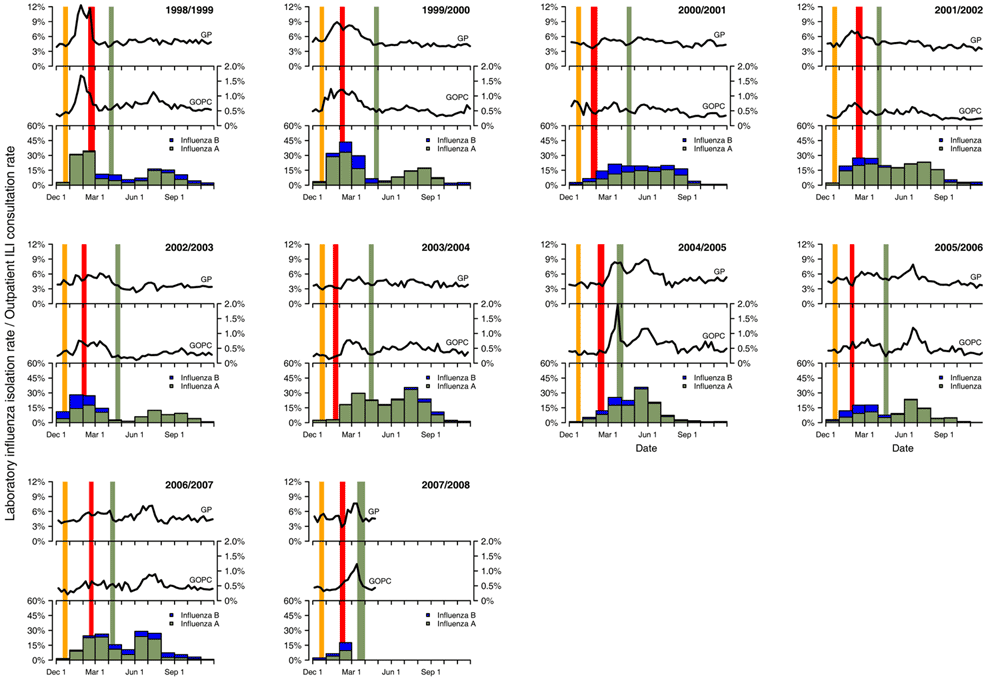

Supplement: Appendix Figure — Weekly influenza-like illness (ILI) consultation rates in sentinel networks of outpatient clinics in the private (GP) and public (GOPC) sectors between December 1988 and March 2008 (lines) and monthly proportions of influenza A and B isolations among all specimens submitted to the Public Health Laboratory of the Department of Health of the Hong Kong Special Administrative Region, People's Republic of China, government (bars); the vertical superimposed bars indicate school holidays at Christmas, Chinese New Year, and Easter. Source: (7). [file 08-0646_app-s1.gif]
